# Effect of Sampling Rate, Filtering, and Torque Onset Detection on Quadriceps Rate of Torque Development and Torque Steadiness

**DOI:** 10.3390/s24134250

**Published:** 2024-06-30

**Authors:** McKenzie S. White, Megan C. Graham, Tereza Janatova, Gregory S. Hawk, Katherine L. Thompson, Brian Noehren

**Affiliations:** 1 Department of Physical Therapy, University of Kentucky, Lexington, KY 40536, USA; mjwh227@uky.edu (M.S.W.); megan.graham@uky.edu (M.C.G.); t.janatova@uky.edu (T.J.); 2 Department of Statistics, University of Kentucky, Lexington, KY 40536, USA; greg.hawk@uky.edu (G.S.H.); katherine.thompson@uky.edu (K.L.T.)

**Keywords:** rate of force development, rate of torque development, signal processing, strength, quadriceps

## Abstract

Quadriceps rate of torque development (RTD) and torque steadiness are valuable metrics for assessing explosive strength and the ability to control force over a sustained period of time, which can inform clinical assessments of knee function. Despite their widespread use, there is a significant gap in standardized methodology for measuring these metrics, which limits their utility in comparing outcomes across different studies and populations. To address these gaps, we evaluated the influence of sampling rates, signal filtering, and torque onset detection on RTD and torque steadiness. Twenty-seven participants with a history of a primary anterior cruciate ligament reconstruction (N = 27 (11 male/16 female), age = 23 ± 8 years, body mass index = 26 ± 4 kg/m^2^) and thirty-two control participants (N = 32 (13 male/19 female), age = 23 ± 7 years, body mass index = 23 ± 3 kg/m^2^) underwent isometric quadriceps strength testing, with data collected at 2222 Hz on an isokinetic dynamometer. The torque–time signal was downsampled to approximately 100 and 1000 Hz and processed using a low-pass, zero-lag Butterworth filter with a range of cutoff frequencies spanning 10–200 Hz. The thresholds used to detect torque onset were defined as 0.1 Nm, 1 Nm, and 5 Nm. RTD between 0 and 100 ms, 0 and 200 ms, and 40–160 ms was computed, as well as absolute and relative torque steadiness. Relative differences were computed by comparing all outcomes to the “gold standard” values computed, with a sampling rate of 2222 Hz, a cutoff frequency in the low-pass filter of 150 Hz, and torque onset of 1 Nm, and compared utilizing linear mixed models. While all combinations of signal collection and processing parameters reached statistical significance (*p* < 0.05), these differences were consistent between injured and control limbs. Additionally, clinically relevant differences (+/−10%) were primarily observed through torque onset detection methods and primarily affected RTD between 0 and 100 ms. Although measurements of RTD and torque steadiness were generally robust against diverse signal collection and processing parameters, the selection of torque onset should be carefully considered, especially in early RTD assessments that have shorter time epochs.

## 1. Introduction

Peak torque and rate of torque (RTD) development are fundamental metrics commonly used to assess maximal and explosive muscle strength. While both metrics contribute valuable insights into a muscle’s capacity for force production, RTD complements maximal strength assessments by providing insights into neural and contractile function that are not captured by maximum strength alone [1,2]. For instance, the initial phase of RTD (<75 ms) is believed to be predominantly affected by neural factors such as motor unit recruitment and discharge rate [3,4], while RTD in the later intervals (>75 ms) has been shown to be more sensitive to the speed-related properties of muscle such as fiber type composition and muscle contractile properties [5,6,7,8]. Rate of torque development has also been associated with a host of functional outcomes, including gait [9,10], landing and running mechanics [10,11], and neural drive [12]. Recognizing the significance of these properties in functional and sport-specific tasks, RTD assessments have gained prominence across populations to measure performance [13,14], monitor rehabilitation [10,15], and understand the mechanisms behind injury and disease [16,17,18,19].

Another metric that provides insight into neuromuscular function is torque steadiness, or the ability to control force over a sustained period of time. While less work has been conducted regarding the clinical implications of torque steadiness, it has been shown that the ability to control force during a steady-state contraction is associated with the ability to control force during functional tasks (e.g., knee flexion excursion during running) and is impaired following injuries and surgeries such as anterior cruciate ligament reconstruction (ACLR) [20]. Furthermore, individuals who received targeted interventions to improve torque steadiness following the failure of conventional physical therapy after an ACLR were able to improve their quadriceps strength and self-reported function [21]. As such, torque steadiness may have different physiological underpinnings than those of maximal and explosive strength and serves as an additional assessment of quadriceps function and should be included in devices and protocols being designed to evaluate muscle performance.

Together, explosive strength and torque steadiness may provide unique insights into knee function and performance. However, there is a lack of methodological standardization in the field that poses challenges in comparing measurements across research sites and patient populations, which may ultimately limit the interpretability and clinical relevance of RTD and torque steadiness measurements. While torque–time signals collected on an isokinetic dynamometer are seemingly straightforward, the intricacies lie in the choices made during signal collection and processing. Signal collection introduces variations through the sampling rates, which have ranged from 100 to 2000 Hz in the literature [3,14,17,22,23] and are often a constraint of commercial dynamometers. In signal filtering, a prevalent approach involves the use of a fourth-order low-pass Butterworth filter [5]. However, the chosen cutoff frequencies used in the low-pass filter span from 3 to 500 Hz [12,24,25,26] and may exert a direct influence on torque–time variables [27]. Another source of variability in RTD measurements is the historical determination of torque onset through set thresholds [6], manual methods [23], or other mathematical algorithms [28]. Given the large variation in methods, standardizing these decisions is imperative to understand the clinical importance of RTD and torque steadiness in control and pathological populations.

Early foundational work has investigated the influence of sampling rate, cutoff frequency [27], and torque onset thresholds [28] on quadriceps RTD measurements in control populations. However, it is possible that more sensitive signal collection and processing techniques may be required in those with pathology compared to control groups. In addition, the impact of different sampling rates and cutoff frequencies on the assessment of torque steadiness has not been thoroughly investigated. Therefore, the primary purpose of this study was to evaluate the influence of sampling rate and signal processing decisions on quadriceps RTD and torque steadiness in controls and in those with a history of ACLR. Through this approach, the findings from this paper aim to determine the feasibility of comparing RTD and torque steadiness outcomes in the literature and underscore the significance of methodological rigor in advancing our understanding of neuromuscular function.

## 2. Methods

### 2.1. Study Design and Participants

The data for this cross-sectional study were collected between April 2022 and September 2023 and were secondary analyses performed from research protocols reviewed and approved by the Institutional Review Board at the University of Kentucky. Participants provided informed consent for the primary studies and secondary data analyses. Twenty-seven participants with a history of a primary ACLR (N = 27 (11 male/16 female), age = 23 ± 8 years, body mass index = 26 ± 4 kg/m^2^) and thirty-two control participants were included (N = 32 (13 male/19 female), age = 23 ± 7 years, body mass index = 23 ± 3 kg/m^2^). Injured participants were eligible to enroll in this study if they had a history of unilateral or bilateral ACLR. Control participants were eligible to enroll in this study if they had no history of lower extremity injury or surgery.

### 2.2. Isometric Strength

Bilateral isometric quadriceps strength was collected at 2222 Hz using a Biodex System 4 isokinetic dynamometer (Biodex Medical Systems Inc., Shirley, NY, USA) and a Delsys Trigno Analog sensor (Delsys Inc., Natick, MA, USA). The sampling rate of 2222 Hz was inherent to the Trigno device adapter specifications. Each participant was secured in the dynamometer with straps over the shoulders, lap, and thigh and the knee positioned to 90° of flexion. Participants completed a single submaximal practice trial to allow for familiarization [5] and four test trials at maximal voluntary isometric contraction. They were instructed to kick as hard and fast as possible and maintain this effort for a total of five seconds in each trial. Participants were given 30 s of rest between each trial and were provided strong verbal encouragement and real-time visual feedback to encourage maximal effort during all testing contractions.

### 2.3. Signal Processing

The raw data per trial were processed with custom-written software using MATLAB version 2023b (MathWorks, Inc., Natick, MA, USA). Data were converted to torque units (Nm), and the offset from the Biodex system was removed to set a baseline of zero. To determine the effect of sampling rate on the RTD and torque steadiness, ground truth data (2222 Hz) were downsampled to approximately 100 and 1100 Hz (e.g., 101 and 1111 Hz). These sampling rates (100, 1100, and 2222 Hz) reflect commonly used sampling rates of commercial dynamometers, analog-to-digital converters, and previous recommendations [3,5,22]. To determine the effect of cut-off frequency on RTD and torque steadiness, all data were filtered using a fourth-order, low-pass, zero-lag Butterworth digital filter with a range of cutoff frequencies including 10, 50, 100, 150, and 200 Hz. This range of cutoff frequencies was chosen based on previously published works [26,27,29,30,31].

**Figure 1:**
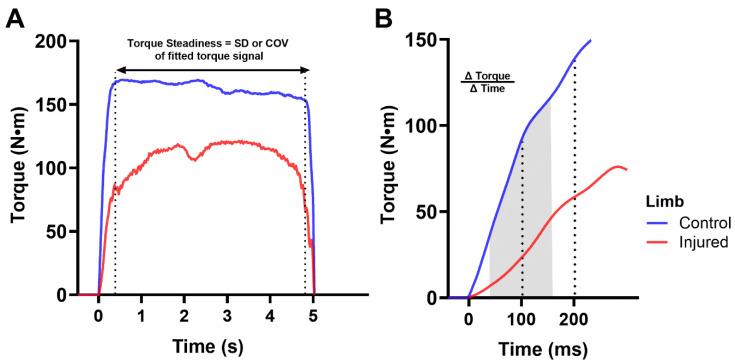
Rate of torque development and torque steadiness outcome measures. Control and injured limbs are represented by blue and red lines, respectively. Torque steadiness was measured as absolute and relative in the region of the torque–time signal between the dotted lines (Panel **A**). Absolute torque steadiness was measured as the standard deviation (SD) of the difference between the participant’s torque signal and a 2nd-order polynomial fitted torque signal during the plateau interval shown between the vertical dotted lines. Relative torque steadiness was measured by the coefficient of variation (COV) or where the absolute torque steadiness was divided by the mean torque output. Rate of torque development (Panel **B**) was measured between 0 and 100 ms (RTD100), 0 and 200 ms (RTD200), and 40 and 160 ms (RTD40–160 ms). RTD40–160 ms is indicated by the shaded region and represents the most linear region of the torque–time curve. Abbreviations: SD, standard deviation; COV, coefficient of variation.

RTD outcomes were measured as torque onset to 100 ms (RTD100) and 200 ms (RTD200), representing early and late RTD, and between 40 and 160 ms (RTD40-160) as an alternative to the commonly assessed RTD measures between 20 and 80% of peak torque (Figure 1). The 40–160 ms time interval was chosen to minimize the variability that can be introduced by different approaches to measuring peak torque, while avoiding the variability in RTD measurements that is associated with different thresholds for detecting torque onset. Furthermore, RTD40-160 reflects the steepest linear region of the initial rise in torque.

**Figure 2:**
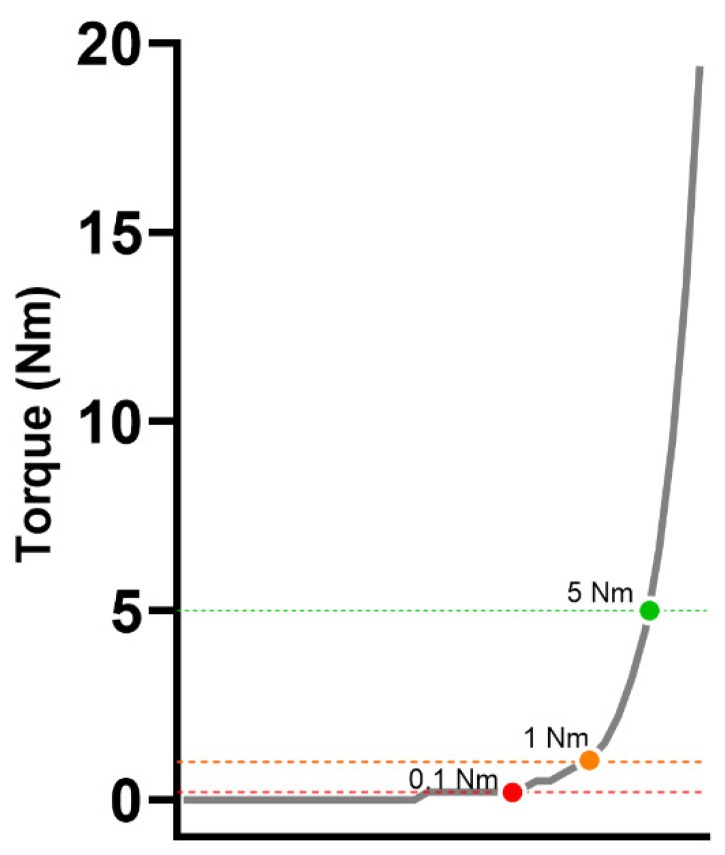
Representative thresholds for detecting torque onset. Thresholds of 0.1, 1, and 5 Nm were used to detect torque onset.

Torque onset was determined when the torque signal reached thresholds of 0.1, 1, and 5 Nm (Figure 2). RTD outcomes were computed for each of the four trials and averaged.

To measure torque steadiness, we first identified the rise and fall-off intervals of the torque signal by measuring the local slope minima and maxima on the derivative of the signal for all combinations of sampling rates and cut-off frequencies listed above. The time in which 20% of these local slope minima and maxima occurred defined the start and end of the rise and fall-off intervals (Figure 1) [20]. Once the interval was defined, a 2nd-order polynomial was fitted to represent the target or “ideal” torque. Torque steadiness was measured as absolute and relative, where absolute was measured as the standard deviation of the difference between the participant’s torque signal and polynomial fitted “ideal” torque signal during the plateau interval (Figure 1A, Equation (1)), and relative was measured by the coefficient of variation or where the absolute torque steadiness was divided by the mean torque output [20].

Absolute torque steadiness = SD(torque_subject_ − torque_polynomial_) (1)

Relative torque steadiness = absolute torque steadiness/mean(torque_subject_) (2)

The following parameters were taken as the baseline “gold standard” based on clinical expertise and previously published guidelines: sampling rate of 2222 Hz, torque onset of 1 Nm (only used for RTD outcomes), cutoff frequency of 150 Hz [23]. For each participant, the relative difference between each RTD and torque steadiness outcome and the gold standard were calculated.

### 2.4. Statistical Analyses

R 4.3.0 (R Foundation for Statistical Computing; Vienna, Austria) was used to create an analysis data set (e.g., calculate relative differences in outcomes) and produce figures; SAS 9.4 (SAS Institute Inc.; Cary, NC, USA) was used for mixed-model analyses and confidence interval calculations. For each of the five outcomes (RTD100, RTD200, RTD40-160, and absolute and relative torque steadiness), data from a single limb of n = 59 participants were analyzed. For patients in the injured group, the limb with an ACLR was used; for control patients, all right limbs were used. For this work, relative differences in each of the five outcomes were analyzed separately (each with a single overall mixed-model fit and hypothesis test), and clinical significance was determined as exceeding a +/−10% deviation from the gold standard value for each respective outcome. A +/−10% deviation was chosen based on the understanding that a limb-symmetry index of at least 90% is a commonly used clinical threshold indicative of adequate quadriceps strength used in return-to-sport criteria following ACLR [32,33]. Falling below this threshold suggests the injured limb may not safe to return to sport and may be at an increased risk for reinjury.

To model the relative effects of changes in the values of the parameter settings from the gold standard settings, a linear mixed model was fitted for each of the five outcomes listed above. Prior to analysis, plots were used to assess normality at each parameter setting; no skewness was identified so no variable transformations were needed. Patient-level random effects helped model the correlation between observations from the same patient at different settings, while the different combinations of scan settings for control and injured limbs were included as a fixed effect. For RTD outcomes, this was formulated as a four-way interaction between sampling rate, cutoff frequency, torque onset, and limb; for torque steadiness outcomes, this was formulated as a three-way interaction between sampling frequency, cutoff frequency, and limb. All mixed models contained the full factorial of lower-order interactions and main effects.

**Figure 3:**
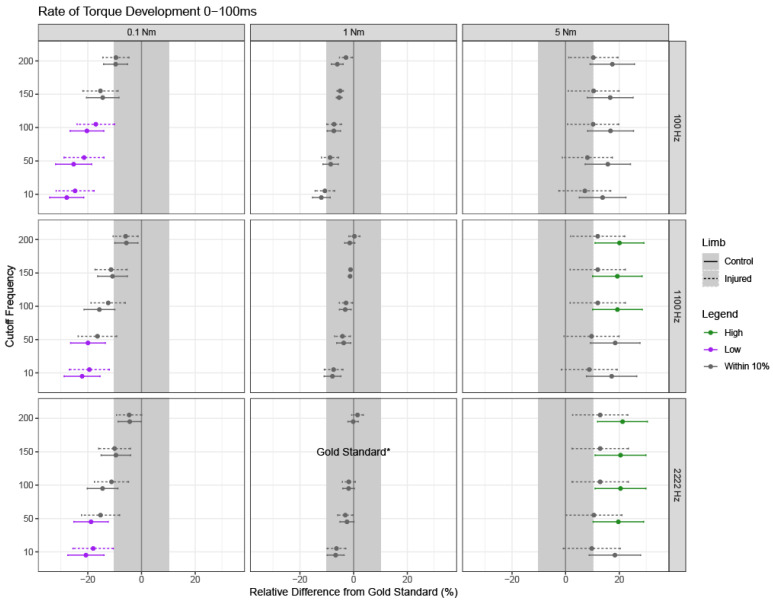
Rate of torque development between 0 and 100 ms (RTD100). RTD100 estimates and 95% confidence intervals for each limb are shown for combinations of cutoff frequency used in the low-pass filter, threshold used to detect torque onset, and sampling frequency, as noted by the subpanel labels. Solid lines indicate confidence intervals for healthy limbs; dashed lines indicate confidence intervals for injured limbs. The dot for each confidence interval represents the mean value. Green and purple confidence intervals demonstrate clinically significant overestimation or underestimation from the gold standard, respectively; grey confidence intervals are not clinically different from the gold standard (less than a +/−10% relative difference). Note that the gold standard settings were a 150 Hz cutoff frequency used in the low-pass filter, a 1 Nm threshold for detecting torque onset, and a 2222 Hz sampling frequency, and thus, no intervals or estimates exist for these settings (indicated by “gold standard*” on the bottom row, middle column).

**Figure 4:**
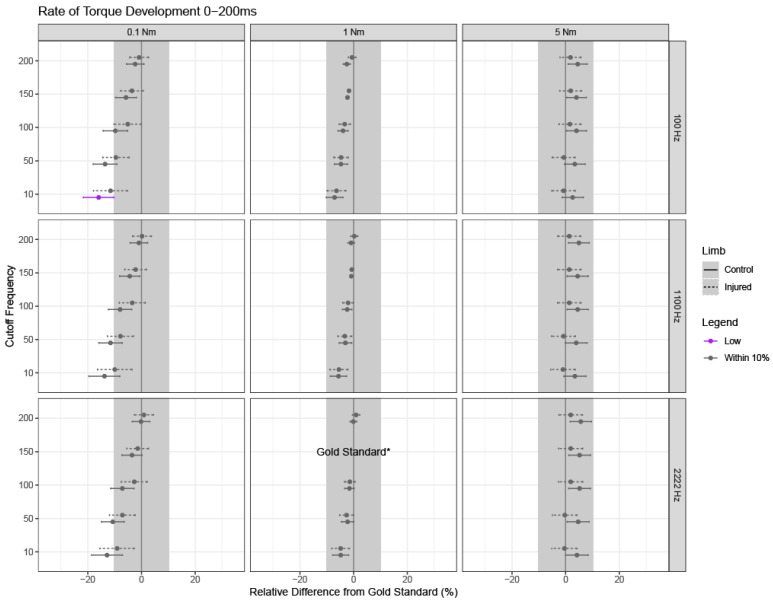
Rate of torque development between 0 and 200 ms (RTD200). RTD200 estimates and 95% confidence intervals for each limb are shown for combinations of cutoff frequency used in the low-pass filter, threshold used to detect torque onset, and sampling frequency, as noted by the subpanel labels. Solid lines indicate confidence intervals for healthy limbs; dashed lines indicate confidence intervals for injured limbs. The dot for each confidence interval represents the mean value. Purple confidence intervals demonstrate clinically significant underestimation from the gold standard; grey confidence intervals are not clinically different from the gold standard (less than a +/−10% relative difference). Note that the gold standard settings were a 150 Hz cutoff frequency used in the low-pass filter, a 1 Nm threshold for detecting torque onset, and a 2222 Hz sampling frequency, and thus, no intervals or estimates exist for these settings (indicated by “gold standard*” on the bottom row, middle column).

**Figure 5:**
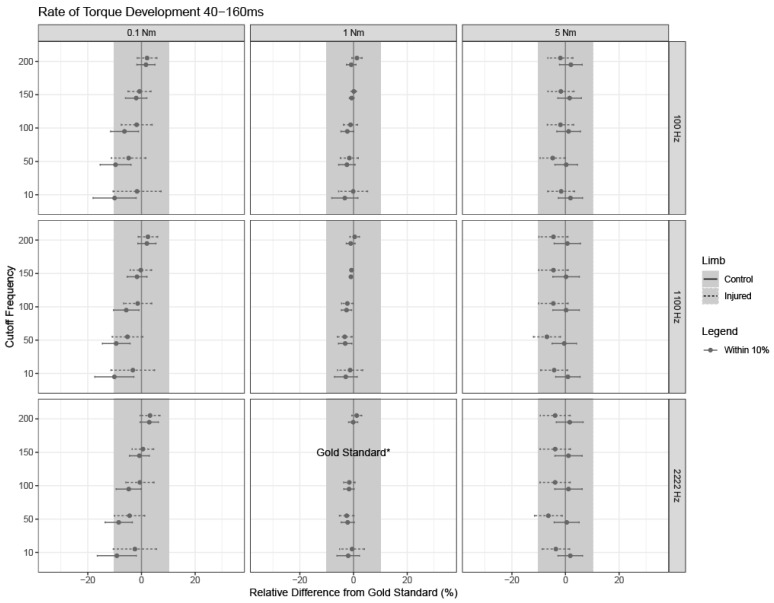
Rate of torque development between 40 and 160 ms (RTD40–160). RTD40-160 estimates and 95% confidence intervals for each limb are shown for combinations of cutoff frequency used in the low-pass filter, threshold used to detect torque onset threshold, and sampling frequency, as noted by the subpanel labels. Solid lines indicate confidence intervals for healthy limbs; dashed lines indicate confidence intervals for injured limbs. The dot for each confidence interval represents the mean value. Grey confidence intervals are not clinically different from the gold standard (less than a +/−10% relative difference). Note that the gold standard settings were a 150 Hz cutoff frequency used in the low-pass filter, a 1 Nm threshold for detecting torque onset, and a 2222 Hz sampling frequency, and thus, no intervals or estimates exist for these settings (indicated by “gold standard*” on the bottom row, middle column).

**Figure 6:**
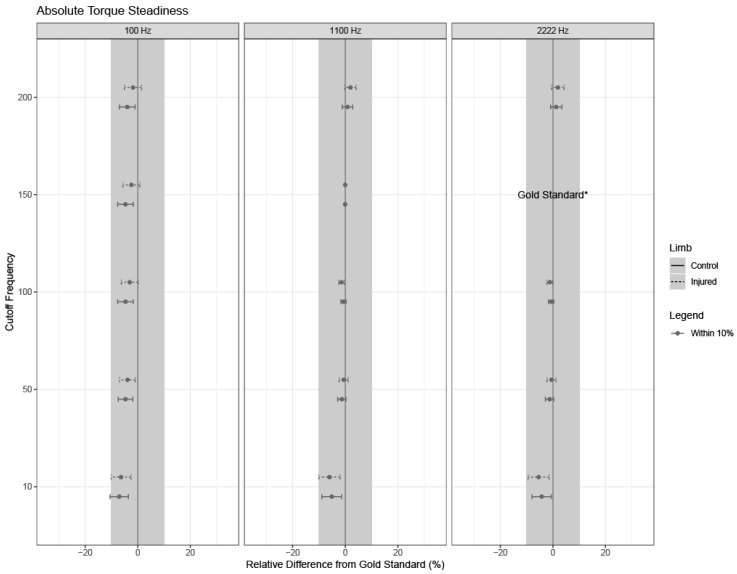
Absolute torque steadiness. Estimates and 95% confidence intervals for each limb are shown for combinations of cutoff frequency used in the low-pass filter and sampling frequency, as noted by the subpanel labels. Solid lines indicate confidence intervals for healthy limbs; dashed lines indicate confidence intervals for injured limbs. The dot for each confidence interval represents the mean value. Grey confidence intervals are not clinically different from the gold standard (less than a +/−10% relative difference). Note that the gold standard settings were a 150 Hz cutoff frequency used in the low-pass filter and a 2222 Hz sampling frequency, and thus, no intervals or estimates exist for these settings (indicated by “gold standard*” on the right column).

**Figure 7:**
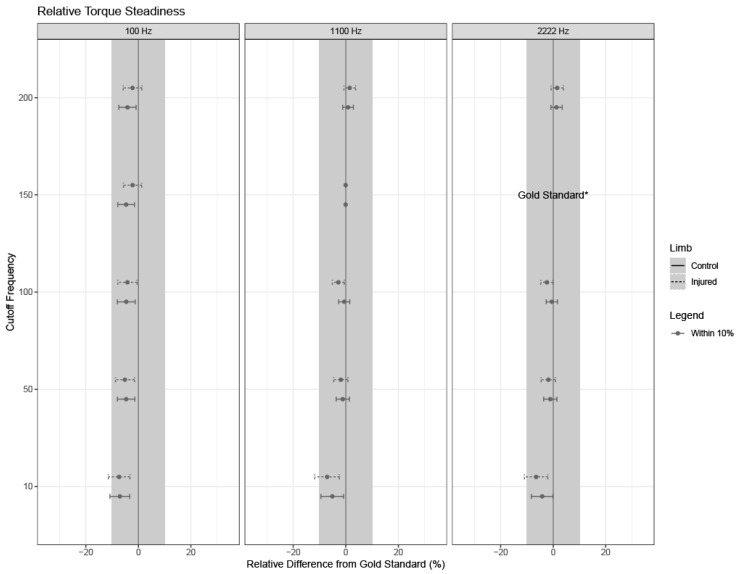
Relative torque steadiness. Estimates and 95% confidence intervals for each limb are shown for combinations of cutoff frequency used in the low-pass filter and sampling frequency, as noted by the subpanel labels. Solid lines indicate confidence intervals for healthy limbs; dashed lines indicate confidence intervals for injured limbs. The dot for each confidence interval represents the mean value. Grey confidence intervals are not clinically different from the gold standard (less than a +/−10% relative difference). Note that the gold standard settings were a 150 Hz cutoff frequency used in the low-pass filter and a 2222 Hz sampling frequency, and thus, no intervals or estimates exist for these settings (indicated by “gold standard*” on the right column).

If the overall model was significant, relevant pairwise differences and corresponding 95% confidence intervals were calculated for each relative difference from the gold standard using Fisher’s Least Significant Differences (LSD). Confidence intervals are shown for each outcome, limb type, and parameter setting, where confidence intervals with bounds outside of +/−10% from the gold standard settings are of clinical significance (Figure 3, Figure 4, Figure 5, Figure 6 and Figure 7).

Likelihood ratio testing and Akaike Information Criterion (AIC) were used to select an appropriate covariance structure in each case, and Kenward–Roger adjustment was used to correct for negative bias in the standard error and degree of freedom calculations induced by small samples [34,35,36]. In each case, models with different covariance structures were compared by likelihood ratio testing, when possible, as well as AIC; the covariance structure producing the lowest AIC was selected to fit the data best, with likelihood ratio testing used to confirm the optimal complexity level of this structure [37]. This covariance selection process was performed prior to the overall hypothesis test for the mixed model. For each mixed model described above, a combination of visual residual plots and formal testing (i.e., Shapiro–Wilk test for normality of the residuals) was used to evaluate all model assumptions. Across all analyses, a *p*-value less than 0.05 was considered statistically significant.

## 3. Results

Our analyses revealed statistically significant differences across all measures of RTD and torque steadiness, as determined by the mixed-model overall F-tests (*p* < 0.001). Despite these differences, confidence intervals for all RTD and torque steadiness outcomes overlapped between the injured and control limbs, irrespective of varying sampling rates, cutoff frequencies, and torque onset detection parameters. This suggests RTD and torque steadiness outcomes can be standardized regardless of injury status.

### 3.1. Early Rate of Torque Development: Torque Onset to 100 ms

Average RTD100 was clinically significant across different torque onset detection threshold values. When using a 0.1 Nm threshold to detect torque onset, clinically relevant differences were noted across all sampling frequencies (Figure 3, left column). Specifically, with a 0.1 Nm torque onset threshold and data sampled at 100 Hz, RTD100 was underestimated by, on average, 22.8% when cutoff frequencies under 100 Hz were used in the low-pass filter, considering both limbs and cutoffs of 10, 50, and 100 Hz and based on the mixed-model results. Similarly, RTD100 was underestimated by, on average, 18.8% when an onset of 0.1 Nm was used and the data were sampled at 1100 and 2222 Hz.

When using a 1 Nm threshold to detect torque onset, no clinically significant differences relative to the gold standard were observed for any combination of cutoff frequencies and sampling rates (Figure 3, middle column).

A 5 Nm threshold for detecting muscle onset did not introduce clinically significant differences when data were sampled at 100 Hz (Figure 3, right column). However, when data were sampled at 1100 Hz, the average RTD100 was most affected at cutoff frequencies above 100 Hz, resulting in a 15.8% overestimation on average. Similarly, when data were sampled at 2222 Hz, RTD100 was affected by all cutoff frequencies other than 10 Hz, resulting in a 16.4% overestimation on average. Despite the confidence intervals for the control and injured limbs largely overlapping, this overestimation only reached clinical significance in the control limb.

### 3.2. Late Rate of Torque Development: Torque Onset to 200 ms

RTD200 was largely stable across all combinations of sampling rates, cutoff frequencies, and torque onset thresholds and deviations in RTD200 were minimized when using a 1 Nm threshold for detecting torque onset (Figure 4, middle column). The only combination of parameters to result in a clinically relevant difference for RTD200 was a sampling rate of 100 Hz, a cutoff frequency of 10 Hz, and an onset threshold of 0.1 Nm, which resulted in a 13.7% underestimation.

### 3.3. Rate of Torque Development between 40 and 160 ms

RTD40-160 was stable across all combinations of sampling rates, cutoff frequencies, and torque onset thresholds. Similarly to RTD100 and RTD200, an onset detection of 1 Nm minimized the effects of using different sampling rates and cutoff frequencies when compared to the gold standard values.

### 3.4. Torque Steadiness

No combinations of sampling rates, cutoff frequencies, and torque onset thresholds introduced clinically significant differences in absolute or relative torque steadiness compared to the gold standard values (Figure 6 and Figure 7).

## 4. Discussion

Rate of torque development has been increasingly used over the past decade as a valuable indicator of muscular fatigue, functional capacity, and explosive strength [26,38,39]. However, diverse methodologies in signal collection and processing have been utilized throughout the literature. As such, the primary aims of this study were to elucidate the impact of sampling rate, the choice of cutoff frequency with low-pass filtering, and the threshold for detecting torque onset on RTD measurements in both control and injured limbs. Although our analyses revealed statistically significant differences in RTD and torque steadiness across various parameter combinations, these differences did not differ between control and injured limbs. Importantly, while statistical significance was achieved, clinically relevant deviations (+/−10%) from the gold standard appeared seldomly, especially in outcomes with larger time intervals (RTD200, RTD40-160, torque steadiness), thereby suggesting that a more tolerant approach to comparison across research studies for outcomes with longer time epochs (>100 ms) could be used. This has important implications in the device design of sensors to evaluate these parameters of quadriceps function and for the design of protocols using different devices (e.g., isokinetic dynamometers, signal amplifiers).

Of the parameters tested, the threshold used to detect torque onset introduced the most variability in RTD outcomes. Early RTD (RTD100) emerged as the outcome most susceptible to variations in torque onset detection thresholds, which deviated, on average, by 13.2% (in absolute value) from the gold standard (Figure 3) using 0.1 Nm and 5 Nm thresholds (Figure 3). Early RTD may be more susceptible to variation when compared to late RTD due to the noise that is present at the start of the collection and in the initial rise of the signal. Additionally, a 100 ms interval is the shortest epoch assessed in the current study, which means that any noise in the signal during that time window can have a larger impact on RTD values. For example, smaller variations in torque onset detection can lead to relatively greater variation in early RTD compared to late RTD. Together, these findings highlight the importance of carefully considering the torque onset threshold in processing procedures, especially for early RTD or measurements taken from shorter time epochs during the initial rise of the torque–time signal. Thompson et al. previously reported a comparison of an automatic versus a manual approach for identifying muscle contraction onset [28]. While automatic thresholding was found to be more reliable than manual methods, a single automatic threshold of 4 Nm was utilized, and the study was performed on the ankle plantarflexors [28]. We further extended this by assessing multiple automated thresholds of 0.1, 1, and 5 Nm for detecting muscle contraction onset in the quadriceps and found that automatic thresholding does indeed impact RTD outcomes, particularly when low or high thresholds are used (0.1 or 5 Nm) and especially for RTD100. However, further work is still needed to refine the automatic thresholding technique by developing standardized tools for preprocessing approaches even before identifying the automatic threshold. This includes removing noise and setting the baseline to zero in the same manner across research studies to ensure the automatic thresholding will be implemented on data that have been pre-preprocessed with the same methods. Given the variability in torque–time data, developing standardized criteria as a field would be greatly beneficial to aid in reproducibility and comparison across research sites.

Interestingly, we observed a minimal impact of sampling rates on the variability in RTD outcomes, particularly when using a 5 Nm threshold for torque onset. For instance, regardless of the sampling rate and cutoff frequency used, relative differences from the gold standard exhibited overlapping confidence intervals across all sampling frequencies when a 5 Nm threshold was set (Figure 3, Figure 4 and Figure 5 right columns). In other words, the width of the confidence intervals and the relative differences were stable across the different sampling frequencies (e.g., rows) for a given threshold for detecting muscle contraction onset, particularly for a threshold of 5 Nm. Moreover, when compared to the gold standard values, the influence of sampling rate on RTD was minimal. Our results suggest that the use of a commercial dynamometer that samples at 100 Hz may be acceptable if the torque onset threshold is 1 or 5 Nm for RTD100 and RTD200 measurements. For higher sampling rates such as 1100 or 2222 Hz, a 1 Nm threshold for detecting torque onset is recommended for RTD100 given that clinically relevant differences were introduced when using 0.1 or 5 Nm threshold with varying cutoff frequencies in the low-pass filter. It should be noted that although the injured and control limbs had overlapping confidence intervals, clinically relevant differences were only introduced in the control limb where RTD100 was overestimated (Figure 3 right panels). It is possible that a higher threshold for detecting muscle onset captures a steeper part of the torque–time curve in control limbs, which can generate a more rapid initial torque–time signal compared to the more gradual signal in injured limbs. Future studies could focus on directly parsing this out by assessing even higher thresholds (e.g., above 5 Nm) and shorter time epochs between control and injured populations.

Filtering cutoff frequencies introduced the most variability across varying torque onset detection thresholds and sampling rates, as shown by the deviation in estimates for 0.1 Nm versus the consistency in estimates for 5 Nm (Figure 3, Figure 4 and Figure 5). Higher cutoff frequencies used in the low-pass filter were able to overcome some of the variability introduced by sampling rate and torque onset. For example, cutoff frequencies under 100 Hz introduced clinically significant differences from the gold standard across all sampling rates for RTD100. For the most part, the longer time epoch introduced with RTD200 overcame the variability from cutoff frequency at 0.1 and 5 Nm seen in RTD100. Similarly to torque onset thresholds, early RTD (shorter epochs) appears to be the most affected, so careful consideration of cutoff frequencies should be considered when implementing a study or comparing studies that utilize different filtering parameters. These findings are similar to those of Thompson et al., who has previously suggested a minimum of 150 Hz cutoff frequency used in the low pass filter for early RTD (~50 ms) [27]. However, methodological differences exist in measurements between the current study and that of Thompson et al., as Thompson investigated instantaneous RTD at 50 ms rather than the overall change in slope within the time epoch (0 to 50 ms) [27]. This is an important consideration as instantaneous RTD may minimize the effect of the slope on the outcome variables and may capture a different aspect of quadriceps function than traditional measurements of RTD that consider more of the torque–time signal. Thompson et al. also suggested that a wide range of cutoffs (≥20 Hz) can be utilized for late RTD (200 ms) [27]. Despite the difference in the measurements of late RTD between Thompson et al. and the current study (instantaneous vs. 0–200 ms), it appears that late RTDs or those of longer time epochs are less susceptible to differences in the cutoff frequencies used in the low-pass filter.

While RTD100 and RTD200 have been commonly used in the literature, RTD40-160 appears to overcome the variability introduced by signal collection and processing decisions. If limited by equipment, sensors, and signal processing techniques, RTD40-160 can serve as a robust measure that is less susceptible to the variability introduced by sampling rate, the cutoff frequencies used in the low-pass filter, and thresholds for determining muscle onset compared to RTD100 and RTD200. RTD40-160 is likely more robust because it occurs after the variability that would be introduced by the threshold for determining muscle onset and shorter time epochs, while also encompassing a steeper part of the torque–time curve.

There are several considerations to consider when interpreting the results of the current study. First, RTD and torque steadiness outcomes change throughout rehabilitation following joint injury. Investigating whether more sensitive methodological considerations are necessary to capture differences throughout the time course of recovery warrants further investigation. While we investigated an array of sampling rates, cutoff frequencies used in the low-pass filter, and thresholds for detecting muscle onset, additional values have been explored. Our primary goal was to determine reproducibility and comparison across research sites that utilize different collection and processing techniques. However, future work would benefit from identifying the optimal sampling rate, cutoff frequency, and threshold for detecting muscle onset that minimizes variability in the outcome measures. Furthermore, utilizing electromyography may overcome the variability introduced by using a set threshold for determining muscle onset, as it can provide a more precise indication of muscle onset based on activation, potentially leading to more accurate RTD measurements. The shortest time epoch assessed in this study was 100 ms, but shorter time epochs (e.g., <75 ms) are of interest due to their indication of neural drive [3,4]. Future research should explore signal collection and processing methods that address variability in shorter time epochs to improve the translation and validation of RTD as an indicator of neural function. In the current study, all right limbs of the control group were used as a comparison to the injured limb as we did not expect control limbs (right vs. left) to require different collection and processing techniques. Rather, we were interested in determining if there was a difference in control versus injured limbs. Future work may investigate if the right and left limbs of a control group are influenced by different collection and processing techniques. Lastly, it is possible that individuals with other pathologies known to impact quadriceps function beyond ACLR (e.g., elderly individuals) require more sensitive signal collection and processing techniques, which warrants further investigation.

Overall, our study highlights the importance of considering standardized methodologies to improve the reproducibility and clinical understanding of RTD and torque steadiness outcomes. While RTD and torque steadiness measurements exhibit resilience to various signal collection and processing decisions, careful consideration of torque onset detection thresholds, particularly in early RTD assessments with shorter time epochs, is important to ensure accurate interpretation and comparisons across research studies. Furthermore, these results provide guidance for future sensor development and postprocessing algorithms used to understand quadriceps function. Importantly, our findings suggest that variations in signal collection and processing methods can be present without significantly impacting the clinical relevance of the results. This flexibility in sensor device design and protocol implementation is crucial for understanding performance and disease mechanisms in populations with pathology, supporting innovation and the broader application of quadriceps function in both research and clinical settings.

## Data Availability

Data are contained within the article.
